# Supplementary material for: Same Brain, Different Look?—The Impact of Scanner, Sequence and Preprocessing on Diffusion Imaging Outcome Parameters
Source: J Clin Med. 2021 Oct 27;10(21):4987. doi: 10.3390/jcm10214987 (PMC8584364; doi:10.3390/jcm10214987)
Supplement: Supplementary file 1 [file jcm-10-04987-s001.zip › jcm-1386163-supplementary.pdf]

Supplementary

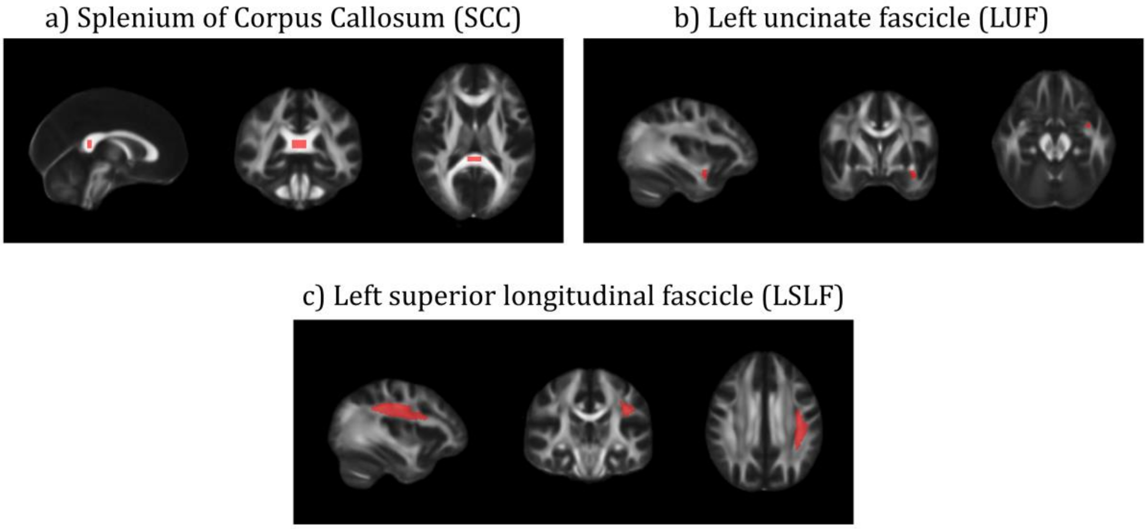

Figure S1. ROIs selected for ROI-based analyses.

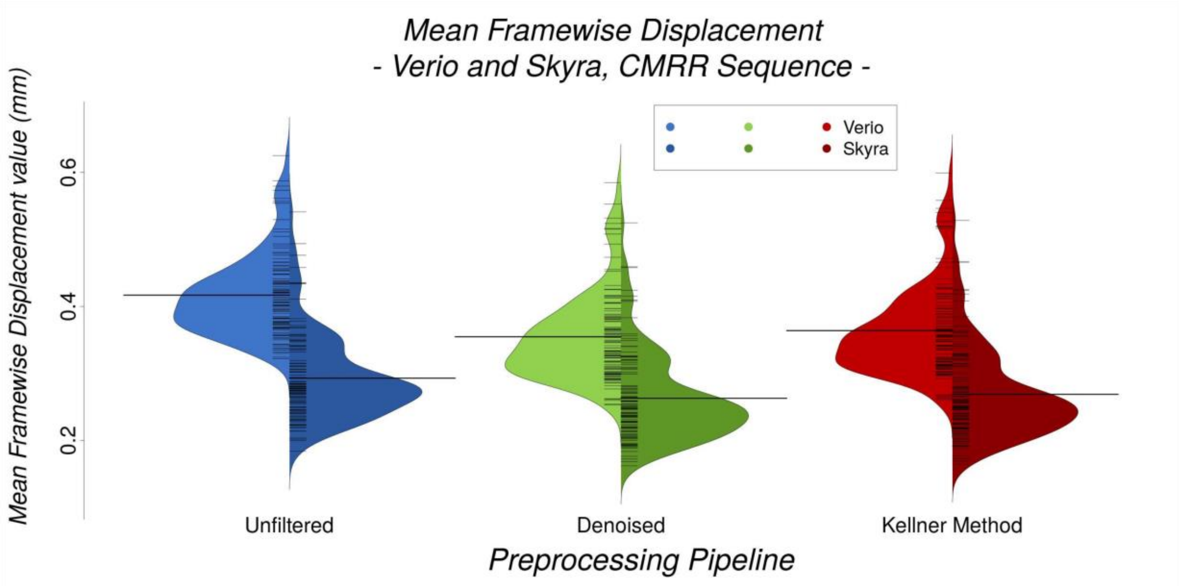

Figure S2. Motion effects quantified as mean frame-wise displacement (Verio and Skyra, CMRR sequence, n = 115) differ between preprocessing pipelines

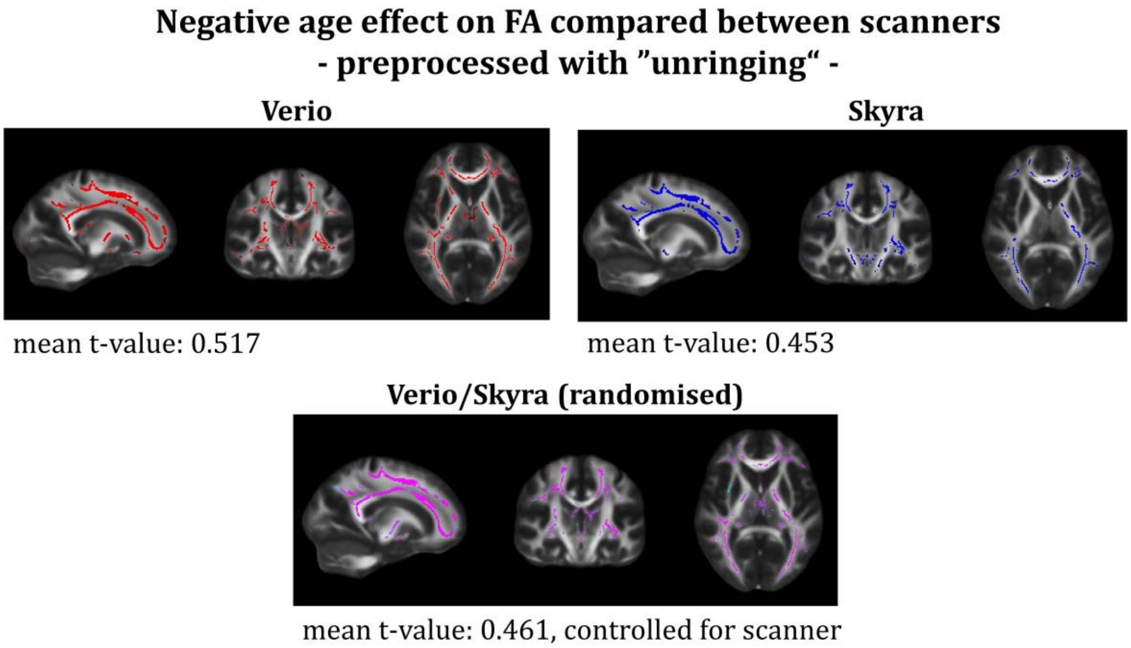

Figure S3. TBSS results of the negative age effect on the whole brain WM skeleton compared between scanners.
